# Foods for Plant-Based Diets: Challenges and Innovations

**DOI:** 10.3390/foods10020293

**Published:** 2021-02-01

**Authors:** Alexandra Alcorta, Adrià Porta, Amparo Tárrega, María Dolores Alvarez, M. Pilar Vaquero

**Affiliations:** 1 Instituto de Ciencia y Tecnología de Alimentos y Nutrición (ICTAN-CSIC), 28040 Madrid, Spain; a.alcorta@ictan.csic.es (A.A.); a.porta@ictan.csic.es (A.P.); mayoyes@ictan.csic.es (M.D.A.); 2 Instituto de Agroquímica y Tecnología de Alimentos (IATA-CSIC), 46980 Paterna, Spain; atarrega@iata.csic.es

**Keywords:** meat alternatives, plant-based dairy, fish alternatives, vegan, vegetarian, flexitarian, plant-based diet, consumer perception, fortified food, nutrients

## Abstract

Plant-based diets have become popular as a means of reducing the environmental footprint of the diet and promoting human health and animal welfare. Although the percentages of vegetarians and vegans are low compared to omnivores, their numbers have increased significantly in the last years. The use of non-animal food products other than meat alternatives is also increasing and this tendency constitutes an opportunity for the food industry. In this review, we present that plant-based meat and milk alternatives are consolidated but that there is a niche for egg, seafood alternatives, and new products which may not resemble any traditional animal food. However, not all animal food substitutes are sustainable and some of them are even ultra-processed. In addition, there are concerns on safety and labeling, and consumers demand clear information and regulation. The challenges in this field are connected with food design and technology, sensory science, nutrition, and dietetics. Moreover, adequate selection and combination of foods is important in order to achieve consumer acceptance while preventing nutritional deficiencies in those who choose this type of diet.

## 1. Introduction

Vegetarianism, veganism and the adoption of a plant-based diet are growing trends across Western countries. Although plant-based diets are often equated with vegetarian diets, they consist of different eating patterns. The term, plant-based, is wider as it focuses on consumption of foods primarily from plants (fruit, vegetables, nuts, oil, whole grains, and legumes), but can include small quantities of food from animal origin such as milk, eggs, meat and fish [1]. Those who follow a plant-based diet might choose to substitute animal products for vegetable options, without permanent restriction of animal foods. In addition, some authors consider that the Mediterranean Diet is mainly a plant-based diet [2].

Commonly reported reasons to follow a plant-based diet include concerns for health, environment, animal welfare, rejection of meat, and religious beliefs [3]. Different reports present the higher environmental impact of meat from ruminants compared to grains, fruit and vegetables [4,5,6]. In terms of population subgroups likely to choose plant-based diets, young adults and women have been found to be less resistant to reducing or avoiding meat consumption [7,8].

## 2. Consumer and Market Trends

The number of people following plant-based diets is increasing tremendously, according to different vegan societies and consulting companies. In America, vegans increased by 500%, from nearly four million in 2014 to 19.6 million in 2017 [9]. A national USA survey published in 2018 found that two-thirds of participants had reduced meat consumption in the last three years [10,11]. In the UK, 21% of the population consider themselves flexitarian (vegetarian who eat occasionally food from animals) and 1 in 8 declared being vegetarian or vegan. In Germany, vegetarians increased from 1% in 2005 to 7% in 2018; in Italy the meat-free population has increased by 94.4% from 2011 to 2016, and in Spain, flexitarians increased by 25% in two years [9,12]. Moreover, a global survey in 2019 reported that 40% of consumers are trying to reduce their consumption of animal proteins, while 10% avoided red meat completely [11].

The plant-based meat alternatives global market is projected to increase from USD 1.6 billion in 2019 to USD 3.5 billion by 2026 [13]. The top selling meat alternative products in 2019 were burger (USD 283 million), sausages and hot dogs (USD 159 million), and patties (USD 120 million) [14]. Other data show that meat sales have decreased by 5% from 2015 to 2019 in the US [11].

The plant-based milk alternatives market has also expanded considerably in recent years, more than doubling its sales worldwide from 2009 to 2015, and reaching USD 21 billion [15]. According to Mintel, cow’s milk sales have decreased from USD 19 billion in 2013 to less than USD 16 billion in 2018 [16]. On the contrary, dairy alternatives have increased their sales. According to the Plant Based Foods Association, sales of plant-based yogurts have grown by 55%, plant-based cheeses by 43%, and plant-based creamers by 131% in the US [17].

## 3. Food Products for Vegetarian and Vegan Consumers

Consumers who call themselves vegetarian ingest all type of plant-based food and reject animal food. The vegetarian diet may include eggs, dairy and honey, while the vegan diet does not include any food or derivatives of animal origin.

A variety of meat and milk alternatives are available and are widely accepted and used in vegetarian and vegan diets, while other products like cheese, egg and fish substitutes are in development and will be briefly presented in the innovations section of this review. Table 1 presents description, advantages and disadvantages of all these alternatives.

Meat alternatives are products that resemble meat in their sensory attributes but made from protein sources that do not come from animals. Although insects are considered a meat alternative for their high protein content, they are not suitable for vegetarians and vegans and therefore will not be discussed in this review.

Plant-based proteins are the most common ingredients used to prepare meat alternatives. Soy, wheat gluten and mushrooms are the main ingredients used. Soy is used for its high nutritional quality to prepare protein rich products, such as tofu, obtained from coagulating and pressing soy curds into a compact block. Soy flour is the least processed soy product and is used both in the preparation of soy texturized vegetable protein and soy protein concentrate (70% protein) and isolate (90% protein). Soy meat alternatives, such as texturized vegetable protein, are usually prepared by an extrusion process, which allows for different shapes and sizes of the product [18,19]. Wheat gluten, also called seitan, is obtained during the isolation of starch from wheat flour and is used for its binding, dough-forming and leavening ability. Its cohesive and chewy quality gives the meat-like texture to the products prepared with wheat gluten. Mushrooms are also added to products for their chewiness [18]. Legume proteins from pea, lentil, lupine or chickpea have also been used in the formulation of meat alternatives. Among these, pea-based protein is the most promising. Oilseed proteins from rapeseed and canola can be used as structuring agents when heated, promoting meat-like textures [20].

Mycoprotein is a protein rich product obtained from the mycelium produced by the growth of the fungus *Fusarium venenatum* during fermentation, which can be processed for human consumption. Mycoprotein is mixed with a small quantity of egg albumen, some roasted barley malt extract and water or a natural flavoring is mixed in instead of malt to give a savory character [28]. The filamentous structure of mycoprotein is what gives this product the meat-like texture.

Cultured meat is produced by growing the cells of livestock in a culture medium to recreate the complex structure of animal muscle tissue. Although, technically, the use of animals is necessary to retrieve the animal cells, very few animals are necessary for the production of high quantities of meat due to cell proliferation [31].

Plant-based milk alternatives are water-soluble extracts from legumes (chickpeas, soybeans), cereals (oats, rice), pseudo-cereals (quinoa, teff, amaranth), nuts (almonds, cashew nuts, hazelnuts, walnuts, coconut) or seeds (sesame, sunflower) that resemble cow’s milk and are consumed as a substitute. The milk-like fluids result from breakdown (size reduction) of plant material (cereals, pseudo-cereals, legumes oilseeds, nuts) extracted in water and further homogenization of such fluids, which imitates cow’s milk in appearance and consistency [37]. The main drivers for consumption of these milk alternatives are adopting a vegetarian or vegan diet, and also health reasons, e.g., lactose intolerance or cow’s milk protein allergy [15].

Microalgae are microscopic algae rich in protein, carbohydrates, lipids and other bioactive compounds. Microalgae-derived proteins have complete essential amino acids profiles and their protein content is higher than that of conventional sources, such as meat, poultry and dairy products. In addition, they are a source of polyunsaturated fatty acids. Therefore, microalgae and its derived compounds have been recently used as dietary supplements and sources [57]. Furthermore, microalgae have been recently pointed out by 130 national academies of science and medicine as one of the innovative foods that can bring benefits to human health and climate in the near future [58].

## 4. Challenges

### 4.1. Consumer Challenges

Meat has been in our diet since the beginning of time and has a strong cultural and gastronomic significance. Not surprisingly, many people consider meat to be an important part of the meal both culturally and as an indispensable source of nutrients [11,21,26]. According to Piazza et al. [22] the majority of justifications people give for eating meat are it being natural, normal, necessary and nice (4Ns theory). Meat has also been associated with formal meals, such as restaurant dinners or business meals and is deemed to be more acceptable in these situations compared to meat alternatives. For informal situations, such as eating alone or with one’s family on a weekday, meat alternatives are better accepted [23]. Meat alternatives are, thus, more likely to be accepted by the public when portrayed as a healthy alternative in informal meals (Table 1).

Vegetarian and vegan diets have repeatedly been regarded as inconvenient, products difficult to cook or prepare and their ingredients not always available in stores [11,24]. Contrary to previous findings regarding environmental awareness, meat eaters in the UK do not need to be persuaded by ethical and environmental arguments, as they recognize the benefits of reducing animal product consumption. Most meat-eaters are aware of the benefits of switching to a more vegetable diet but find vegetarianism and veganism inconvenient, expensive or not enjoyable [25]. These aspects prevent purchase and consumption of these products, even when there is an awareness of their environmental and health benefits.

Food neophobia is characterized by a reluctance to consume novel or unfamiliar foods and is common in consumers when first trying plant-based alternatives [4,5,6,59]. It is more easily triggered when consumers are exposed to unfamiliar, processed or different products than the traditional foods they are used to. Early familiarization during childhood is determinant to reduce food neophobia and develop food habits of a variety of traditional and new food items. Meat substitutes derived from soy, legumes and cereals might be a good option to introduce consumers to plant-based diets, as they are less likely to trigger feelings of rejection and suspicion than more unfamiliar alternatives. In this respect cultured meat, which is grown from animal cells in a culture medium, is often viewed as unnatural and raises concerns among consumers related to its safety [32,33,60].

Food neophobia can be partially alleviated through informative and clear labeling. Product perception can be improved by specifying the exact protein source in the ingredients list, confirming a trend toward “clean labeling” desired by consumers. Schouteten et al. [61] found that acceptance of insect-based burgers improved for consumers when they were informed of the ingredients compared to when information was not disclosed. Similarly, when participants are informed in a nontechnical way that emphasizes the final product instead of the production method, the perception of cultured meat improves [33].

A major challenge of plant-based meat alternatives is to recreate the appearance, texture, flavor and mouthfeel of meat products. While vegetarian and vegan consumers are more likely to accept plant-based alternatives that lack meat-like sensory properties, omnivorous and flexitarian consumers prefer alternatives that resemble meat as much as possible [23,62]. According to Michel et al. [23], taste plays the most important role, as some consumers refuse to purchase protein alternatives because they “won’t like the taste”. Furthermore, consumers most frequently associate regular meat with taste in contrast to meat alternatives [23]. This indicates that taste is a major driver in favor of meat, making it a challenge for the food industry to match the flavor of meat alternatives with regular meat. Interestingly, consumers find plant based alternatives more convincing when they imitate processed meat products (hamburgers, sausages, nuggets) than when they imitate unprocessed meats (e. g. steak) [23]. This is likely because the texture of processed meats is easier to replicate than the complex matrix of unprocessed meat. Meat alternatives, therefore, have a better chance if presented in a format that resembles processed meat products such as burgers and sausages.

To mimic the sensory properties of meat, plant proteins require a high degree of processing and manipulation, which modifies the product to such a degree from the original ingredients that it can trigger food neophobia. Clark and Bodgan [5,6] found the main reasons for not increasing the consumption of protein alternatives were being “too processed” and “high in sodium” among the group unlikely to purchase them. Among the group likely to purchase such products, “too many preservatives” was one of the main deterrents.

Milk alternatives have similar sensory challenges to overcome, especially among regular cow’s milk consumers. Cow’s milk has repeatedly been preferred over soy-based alternatives in comparative sensory testing, for both adult and children studies [36,37,38]. Participants in these studies were not regular plant milk consumers. In a study involving regular soy consumers and non-soy consumers, both groups agreed that the taste of soy was the greatest factor in consuming or not consuming soy products, including soy milk [41]. It has been reported that consumers perceived plant-based milk alternatives as more sustainable than dairy products, although the “organic” status also played a significant role in perception [42]. Consumers look for naturally sweetened or no added sugar beverages. McCarthy et al. [43] showed that sugar content in plant-based beverages was the most important attribute, whereas the most important attribute for cow’s milk consumers was fat content. Another study confirms that negative perceptions of cow’s milk are related to high cholesterol, fat and energy content of whole milk [63]. Therefore, the sensorial improvements should be made without increasing the content of fat and sugar beyond acceptable levels taking into account the nutritional composition of the final product.

### 4.2. Sustainability Challenges

The current agriculture and food systems are threatened by pressing issues of the present and the future: population growth, competition for natural resources, climate change, conflicts, crises and food losses and waste. Under these circumstances, our agricultural landscape is in need of a transition to more sustainable food production systems and products. Finding solutions to improve our food systems will help us move towards the Sustainable Development Goals (SDGs) for peace and prosperity for people and the planet, described by The 2030 Agenda for Sustainable Development and adopted by all United Nations Member States in 2015 [64].

Although meat alternatives and other plant-based products (e.g., beverages) are presented as less environmentally harmful, the sustainability gains from these products are still a subject of debate and study. The evidence is clear on the lower environmental footprint of legume production [5,6] but the influence of legumes in reducing greenhouse gas emissions also depends on the management of the agro-ecosystem used (e.g., mono cropping vs. conservation agriculture) [65]. In addition, the post-harvest processing factor may play a role in sustainability.

Cultured meat, also called lab-meat, synthetic meat or in-vitro meat, is still in the early stages of development and its environmental impact in large-scale production is still unclear. Mycoprotein has been found to have a higher global warming impact than chicken and pork, although land use is substantially lower [29]. A life cycle assessment study comparing different meat alternatives found the highest environmental impact for cultured meat and mycoprotein, medium impact for chicken (local-feed), dairy and gluten-based alternatives, and the lowest impact for insects and soy-based alternatives [30].

Last, microalgae require less land area for protein production than animal sources. Algae requires about 2.5 m^2^/kg of protein production compared to 47–64 m^2^ for pork, 42–52 m^2^ for chicken, and 144–258 m^2^ for beef. Furthermore, microalgae are also efficient CO₂ fixing agents. Microalgae are ecologically beneficial with a CO₂ fixation efficiency of about 90%, which is very high compared to that of the terrestrial plants. Genetically modified microalgae have received attention for their potential to enhance production of desired metabolites, but environmental risks must be adequately assessed for open cultivation [55].

In relation to sustainability of meat alternatives, the evidence shows a clear relationship between lower processing requirements and lower environmental impact, while alternatives demanding extensive processing or considerable energy inputs derived from technology see their environmental footprint increased (Table 1).

Generally, it is considered that milk production has a considerable environmental impact compared with milk substitutes [44]. However, among plant-based milks, almond milk has a considerable impact due to its irrigation needs and zinc fertilizer use and transportation to retail store [45]. When producing cheese alternatives, producers should be aware of the environmental costs of palm oil, as most of palm oil is not produced according to the Roundtable on Sustainable Palm Oil certification standard [50].

A closely related challenge is that awareness among the general public of the environmental burden of meat production and consumption is still low [7,66,67,68]. Furthermore, some consumers perceive soy products to have a similar environmental impact to meat [8]. The authors argue that a possible explanation for this is that soy production for livestock feed is a major cause of deforestation in Brazil, which the public confuses with soy for human consumption. Despite these results, public opinion has likely shifted in recent years as a result of consistent efforts by public organisms, institutions and NGOs to raise awareness over the environmental impact of meat and animal products [68].

The current Position of the Academy of Nutrition and Dietetics [69] regarding vegetarian diets includes a statement about sustainability: “… appropriately planned vegetarian, including vegan, diets are healthful, nutritionally adequate, and may provide health benefits for the prevention and treatment of certain diseases. These diets are appropriate for all stages of the life cycle, including pregnancy, lactation, infancy, childhood, adolescence, older adulthood, and for athletes. Plant-based diets are more environmentally sustainable than diets rich in animal products because they use fewer natural resources and are associated with much less environmental damage”.

In this context, public engagement is promoted in different ways. The EAT-Lancet Commission on Food, Planet, Health [70] proposed a Planetary Health Diet, a plant-based diet to feed the growing global population within the boundaries of a safe operating space for food systems. The “Meatless Mondays” initiative to cut back on meat consumption has also received considerable attention in recent years and has been adopted by some public schools in an effort to improve children’s eating habits [71].

### 4.3. Health Challenges

#### 4.3.1. Protein

Proteins are essential for our body to function properly and to maintain our health status, as they are the building blocks for the formation of tissues in the human body, and they are regulators (hormones, enzymes, antibodies, etc.). Proteins are formed by amino acids: essential amino acids, those that the body cannot produce by itself and thus must be obtained from food; and non-essential amino acids, that can be synthesized by the human body [72]. The nutritional quality of food proteins can be defined by their ability to cover the needs in essential amino acids for growth and tissue maintenance. The quality of plant proteins (vs. those from animal sources) has become a very debatable topic due to the increase in consumption of plant products.

Important factors besides from the quantity, is the quality of protein intake in terms of digestibility and amino acid composition. About 30 years ago, the Food and Agriculture Organization (FAO) proposed a reference method for evaluating the quality of dietary protein known as Protein Digestibility Corrected Amino Acid Score (PDCAAS). But in 2013, FAO proposed a new index, the Digestible Essential Amino Acid Score (DIAAS), which reflects, not only the amino acid composition of proteins, but also their bioavailability (digestibility in the small intestine) [73].

Bioavailability and amino acid profile of some plant-proteins such as soy are similar to the ones of eggs. However, some anti-nutrients like phytates, tannins and saponins can affect protein absorption. Lower consumption of proteins, especially lysine and methionine amino acids have been reported in vegetarians compared to omnivores [47,74]. Other studies show that the concentration of the amino acids methionine, lysine, tryptophan and threonine are generally lower in plant-based sources of proteins [73]. Nonetheless, a plant-based diet that is well planned and balanced, in which different amino acids are combined and complemented through different plant-based foods, does not result in protein deficit [73,74].

In addition, as it is well-known, there are mechanical and thermal pre-processing techniques (e.g., roasting, dehulling, blanching, soaking, cooking and sprouting), which can be applied to reduce anti-nutrients such as protease inhibitors, decrease off-flavor, and improve mouthfeel and color. However, some anti-nutrients are very resistant. For example, phytates cannot be destroyed entirely even by heating to 100 °C and a fermentation process that produces phytases that hydrolyse phytates into myo-inositol and phosphate may be more efficient [47]. In this regard, there is a need to develop plant-based food products that contain all essential amino acids or at least most of them and without anti-nutrients that decrease their bioavailability. Moreover, using the complementarity concept in terms of amino acid composition between plant protein sources (for example, grains and legumes eaten together or throughout the day), it will be possible to develop new food products and meat analogs of optimized nutritional and organoleptic qualities.

Nowadays, there are options like plant-based products with highly bioavailable plant proteins like soy. Furthermore, other techniques are being investigated and evaluated such as new microbial fermentation techniques, to increase protein in dairy alternatives, and the use of microalgae as source of plant protein [47] (Table 2).

#### 4.3.2. Vitamin B_12_ (Cobalamin)

Vitamin B_12_ is an essential nutrient as it is necessary for the synthesis of DNA and other several functions. Cobalamin deficiency can damage the nervous system and cause irreversible cognitive disorders like confusion, poor memory and in more severe cases, dementia. Other symptoms include gastrointestinal problems, and megaloblastic anemia [80].

Vitamin B_12_ is synthesized exclusively by microorganisms. Animals acquire this vitamin through the grass, where the bacteria responsible for synthesizing B_12_ live, or by B_12_ fortified feed. In contrast, only a few algae and mushrooms contain vitamin B_12_, thus, making plant-based diets inadequate. Several studies have reported lower vitamin B_12_ status in vegetarians, both lacto-ovo-vegetarians and vegans, compared to omnivorous [69,74,75,76]. Other studies showed a tendency to higher deficiency risk in vegans compared to lacto-ovo-vegetarians, even though the contribution of dairy and eggs to the total dietary vitamin B_12_ is small in such diets [69,86]. Therefore, plant-based diet consumers must ingest B_12_-fortified foods or B_12_ supplements to prevent deficiency.

In addition, other factors can play significant effects. For example, food processing might contribute to deficiency as losses of cobalamin up to 50% can occur during food processing which involves cooking, pasteurization and exposure to fluorescent light. Furthermore, a decrease in the absorption capacity for this vitamin is common in ageing. Thus, factors such as food processing and ageing should be compensated with an increase in cobalamin concentration in food [86]. Some researchers claim that the currently recommended intake levels may not be sufficient for an adequate daily intake, with particular regard to aging and the physiological reduction in absorptive capacity. If the aged individual follows a plant-based diet the provision of adequate and bioavailable amounts of the vitamin should be guaranteed.

Attempts to produce cobalamin supplements from non-animal sources have been made using certain algae (e.g., Chlorella) and cyanobacteria such as Spirulina *(Arthrospira)*, which is known to contain large amounts of vitamin B_12_, though unfortunately, it is biologically inactive in humans [77,80,87]. The same happens with fermented foods (such as tempeh), edible mushrooms and nutritional yeast that cannot be relied upon as adequate or practical sources of the vitamin [69,80].

Nowadays, vitamin B_12_ is obtained by controlled biotechnological processes for pharmacology, supplementation and food fortification applications. Some fortified food includes breakfast cereals, non-dairy milk and yogurt alternatives [80,87]. However, there is no evidence that fortification on its own is enough to achieve recommended daily intakes of cobalamin, as quantities of this vitamin are not high enough, thus making it necessary to consume large amounts of these products to reach the required levels. In this regard, studies have consistently shown that the risk of deficiency is higher for plant-based eaters who do not take B_12_ supplements as food fortification has not shown to be sufficient [75].

Vitamin B_12_ is water soluble, meaning that excess is excreted through urine, avoiding toxicity. Therefore, the food industry should evaluate developing plant-based products that contain higher quantities of active forms of B_12_. The standardization of cyanocobalamin-rich plant foods may be useful in preventing vitamin deficiency while overcoming the frequent lack of supplement use [86].

Concerning B_12_ fortification, it has been suggested that lupin can serve as an alternative substrate for soybeans due to its similar protein content resulting in “lupin tempeh”. In a study performed by Wolkers-Rooijackers et al. [78] *Propionibacterium freudenreichii*, a vitamin B_12_ producing bacterium, was used in co-culture *with Rhizopus oryzae* to produce B_12_-enriched lupin tempeh. A significant increase of vitamin B_12_ content (up to 0.97 μg/100 g) was achieved by fermenting lupin using these two bacteria without affecting other parameters, such as texture and volatile organic compounds. Therefore, these results are promising for vitamin B_12_ fortification of legumes making products with increased nutritional value for a healthy human diet.

In addition, Watanabe et al. [77] provide other alternatives to increase vitamin B_12_ in plant-based foods. The juice of fenugreek (*Trigonella foenum graecum*) leaves can be enriched with B_12_ (12.5 μg/100 mL) by certain lactic fermentations. The addition of *Propionibacteria* to cabbage during sauerkraut production results in higher concentrations of B_12_ (7.2 μg/100 g). Compared to synthetic fortification, fortification with natural vitamin-producing microorganisms is widely recognized as safer, more natural and more environmentally friendly [47].

Another technique is hydroponic cultivation, an emerging technology that enables to grow crops directly in nutrient-rich water, which is an interesting approach to increase vitamin B_12_ in foods. It has been shown that when soybean seedlings are placed in a solution containing 10 μmol/L of B_12_ for 24 h, the leaves contain a significantly higher level of this vitamin (9.8 μg/g fresh weight). Japanese radish sprouts (kaiware daikon) also show significant increases in B_12_ content (1.28 μg/g fresh weight) after the seeds had been soaked for 6 h in a solution containing 200 μg/mL of B_12_. A study performed by Watanabe et al. [77] produced B_12_ enriched lettuce leaves cultivated using hydroponics, suggesting that B_12_-enriched lettuce leaves are an excellent source of B_12_. These results propose that B_12_-enriched vegetables may be of special benefit for vegetarians. Furthermore, data has indicated production of soy yogurt with an enhanced production of cyanocobalamin up to 18 μg/L [77] (Table 2).

#### 4.3.3. Vitamin D

Vitamin D is an essential nutrient for the body and for a healthy status as it is necessary to absorb calcium needed for bone mineralization. In addition, vitamin D influences a large number of metabolic pathways beyond bone metabolism [69] as vitamin D receptors are found in many cell types in the body, thus, being involved in numerous functions such as cellular and immune roles [88]. Therefore, this vitamin is necessary to prevent not only skeletal and muscular alterations, such as osteoporosis and rickets but also other numerous diseases.

Although vitamin D can be obtained through sun exposure by activation of a skin precursor, there appears to be a worldwide epidemic of deficiency [80], since there are many factors that influence cutaneous vitamin D synthesis such as lifestyle, latitude, age, skin pigmentation, type of clothes and sunscreen use [89]. Therefore, dietary intake of this vitamin is needed. There are very few food sources that are naturally rich in vitamin D and these are mostly from animals (oysters, beef, fish, milk and eggs) [80], which are also in its higher bioavailable D_3_ form, compared to the D_2_ form found in plant sources. As a result, plant-based diet consumers might be at risk of vitamin D deficiency.

Lower serum vitamin D levels have been reported in vegetarians and vegans when compared to omnivores, especially when the blood was collected in late winter or beginning of spring and especially in those living at high latitudes where there is less opportunity for sun exposure [61,65,69,73]. Therefore, there is a need for vegetarians to increase their vitamin D intake through sources such as milk and eggs, and fortified foods, which preferably contain the high bioavailable D_3_ form. Vegans fall at a higher risk for deficiency as their only reliable source is sun exposure and some fortified plant-based foods such as breakfast cereals, plant-based drinks and mushrooms.

There is still no consensus regarding the amount of vitamin D that should be ingested due to between-person and population variabilities. It is important to notice that the daily reference intakes are given for periods where sun exposure is low, as in winter, and lately the recommended levels of intake are much higher than in the past. A recent meta-analysis using food-based approaches that incorporates individual data concludes that an intake of ~12 μg/day could prevent vitamin D deficiency (i.e., serum 25-hydroxycholecalciferol < 30 nmol/L) in adults in the absence of sufficient ultraviolet B (UVB) radiation [90].

Therefore, the food industry has the challenge of developing creative plant-based food solutions that cover the gap between current intakes and higher recommended intake values by using good sources of vitamin D, which target vegetarian and flexitarian consumers in order to prevent or revert the deficiency that is being faced in these consumers.

Based on this background, food fortification might be the best option to increase the vitamin D supply to the population compared with vitamin D supplementation (e.g., pharmaceutical pills) [90]. Fortification, including biofortification (increase the natural content of vitamin D in food), of a wider range of foods, which accommodate diversity, is likely to have the potential to increase vitamin D intakes across population groups and consequently minimize the prevalence of vitamin D deficiency. Vitamin D-biofortified eggs are a good example of one of these novel food-based solutions, which together with other vitamin D-containing foods, can play a role in tackling low vitamin D intakes [69,74,79,80].

Even though fortification have shown to contribute to lower vitamin D deficiency in some populations such as children, the problem of fortification with this vitamin nowadays is that it is focused mainly in products suitable for lacto-ovo-vegetarians, such as dairy and eggs but not for vegans, which makes it harder for these population to avoid deficiency. Therefore, this contributes to the food industry challenges of developing new options that target all types of vegetarian populations, including vegans.

Several studies state that biofortification with vitamin D could also embrace the practice of ultraviolet (UV) irradiation of mushrooms and baker’s yeast, which have been shown to stimulate their endogenous vitamin D_2_ content [69,74,79,80]. These foods may be a useful strategy to increase vitamin D intakes for vegetarians. There were various studies performed which increased and maintained serum concentrations of 25(OH)D_2_. However, another study providing UV-irradiated mushrooms as part of a meal for six weeks increased serum 25(OH)D_2_ concentrations in participants, whereas serum 25(OH)D_3_ concentrations decreased, thus, showing no effect on vitamin D status [80]. Therefore, further investigation about UV irradiation of mushrooms and baker’s yeast should be done.

As previously mentioned, D_3_ is the most bioavailable form, which makes plant food a poor source of vitamin D. However, a plant-derived version of D_3_ made by a lichen has been announced [80], which seem promising although more information is needed about it (Table 2).

#### 4.3.4. Iron

The maintenance of adequate iron levels is essential for oxygen transport, energy transport and storage, protein synthesis, among other processes that comprise metabolic functions related to growth, immunity, muscular activity, bone strength and the nervous system [81]. Iron deficiency may lead to iron deficiency anemia, bone resorption, alterations in the immune system, and limitations regarding physical activity [17].

Iron deficiency is one of the most common nutritional deficiencies worldwide and it has been associated with following vegan and vegetarian diets among other factors. However, vegetarians generally consume as much iron as, or slightly more, than omnivores. It should be noticed that the bioavailability of non-heme iron of plant sources is lower than that of heme iron of animal sources (meat, poultry and fish), as the former is easily bound to inhibitors that impair its absorption (polyphenols, fiber, etc.) [74,80,81,87,91]. Therefore, the question is if vegetarians by consuming more iron are able to compensate for its low bioavailability.

Iron status may be similar in vegetarians and nonvegetarians due to several reasons. Non-heme iron can be affected by dietetic inhibitors (phytates, polyphenolics, oxalates) or enhancers (vitamin C, organic acids, citric acids) of iron absorption, thus, being a useful strategy for plant-based eaters to combine non-heme iron with enhancers while avoiding combinations with inhibitors to prevent deficiency [74]. In addition, several studies show that non-heme iron absorption increases when there are low iron stores as in the case of vegetarians and vegans as an adaptative response of the body to achieve adequate iron levels. Non-heme iron absorption can be as much as 10 times greater in iron deficient individuals compared to iron-replete individuals [69], which might be why iron status is adequate in vegetarians and have no risk of iron deficiency anemia [74]. Individuals can also adapt to low intakes of iron over time and can reduce iron losses [69].

Insufficient iron status has been reported in vegetarian women, being menstruation and hormonal contraceptive use the main predictors [82]. Other population groups at risk of iron deficiency are children and anyone experiencing bleeding, such as people with ulcers, malabsorptive disorders, or intense menstrual blood losses [81].

With regard to fortification, several studies have shown that food fortification is a promising strategy for reducing the prevalence of anemia in developing countries. Food vehicles must be designed considering its synergistic effects with iron compounds for effective absorption and bioavailability. In other words, the potential effects of iron enhancers and inhibitors have to be taken into account for increasing the effectiveness of fortification. Common foods that are iron fortified at present include salt, sugar, cereal-based products, milk, and other dairy products [81].

Several approaches are used to increase bioavailable iron in plant foods. These techniques include biofortification, ferritin content enrichment, phytic acid reduction (e-g. adding phytases during baking), microencapsulation of the iron fortificant before adding it to the food vehicle, and ascorbic acid addition [81,83]. These could be interesting approaches for food industries to fortify foods with iron even though the cost-benefit of each technique should be discussed and evaluated. Nevertheless, it should be pointed out that the main iron enhancer is ascorbic acid and the main inhibitor is phytic acid (phytates), and that these interact with iron during digestion [81]. Therefore, it is recommended that foods containing iron inhibitors be eaten in separate meals from those that are rich in iron, and these be preferably consumed together with a source of vitamin C (Table 2).

#### 4.3.5. Omega-3 Fatty Acids

Omega-3 fatty acids are important since they are converted into eicosapentaenoic (EPA) and docosahexaenoic (DHA) acids, which play an essential role in health maintenance as they exert several significant functions at neurologic, cardiovascular, cognitive and immune levels [74,84,87].

There are two essential FAs that are polyunsaturated fatty acids: linoleic acid (LA) and linolenic acid (ALA) which belong to the omega-6, and omega-3 families, respectively. LA is a precursor of arachidonic acid (AA), and ALA of EPA and DHA fatty acids. Furthermore, the eicosanoids derived from the omega-6 or omega-3 pathways have pro-inflammatory, or anti-inflammatory properties, respectively. Therefore, these should be balanced to maintain proper health and functioning of the body [74,80,84].

Vegetarian and vegan diets provide high intakes of omega-6, but are low in omega-3 fatty acids, as the principal dietary source of EPA and DHA is oily fish, which is absent in vegetarian diets, and the rate of conversion of ALA to these two omega-3 FAs is very low [56,69,74,84,87]. Consequently, lower serum levels of EPA and DHA have been reported in vegans and vegetarians [74]. Particularly, in pregnant women and children grown under a vegetarian diet this could have important health consequences [74,84,87], thus, requiring supplemental omega-3 FAs. The European Food Safety Authority (EFSA) recommends a daily intake of EPA and DHA of between 2 g and 4 g to reach claimed effects such as the maintenance of blood pressure and triglyceride levels and intakes of 250 mg a day are sufficient for the maintenance of normal cardiac function [92].

There are plant-based sources of ALA: flaxseeds, hemp seeds, chia seeds, leafy green vegetables (both terrestrial and marine), walnuts, and wheat germ, as well as their derived oils [74,84]. However, due to the inefficient conversion of ALA to EPA and DHA, alternative sources are needed in plant-based diets. For this purpose, cultured microalgae, through which fish acquire them, nowadays represent a growing market [58,80] (Table 2).

For lacto-ovo-vegetarians, there are some products that are commonly biofortified like dairy and eggs by incorporating fish oil or algal to cows’ and hens’ feed [74,85]. Certainly, the use of algal oil will be preferred to that of fish oil for producing eggs considering the ethics of the vegetarian consumer. Furthermore, from a dietetic point of view, it could be possible to maximize the metabolic conversion from ALA to EPA and DHA by increasing intake of ALA and decreasing intake of LA, hence achieving an optimal balance between omega-3 and omega-6 FAs [74].

#### 4.3.6. Calcium

Calcium is the most abundant mineral in the human body. Only 1% of the body’s calcium circulates in the blood and tissues and 99% is stored in the bones and teeth. Each year, approximately nine million people worldwide suffer from fractures due to osteoporosis [72]. Due to bone mineral optimization and bone health, calcium has been a nutrient of concern regarding its deficit. Therefore, many products in the food industry are already fortified with calcium, especially, plant-based products such as plant-based drinks and breakfast cereals.

Less calcium intake has been shown in people who follow a vegetarian diet, than in omnivores [74]. Calcium intake in vegetarians can be up to 25% less than in omnivores and its sources come mainly from fortified plant-based drinks. Therefore, it is recommended for plant-based consumers to choose plant-based foods that are rich in calcium but most importantly, with high bioavailability as well as choosing fortified food products to avoid calcium deficiency and bone health maintenance [69,74,80].

More important than how much calcium is consumed, is how much calcium is absorbed. Calcium absorption from foods depends on physiological aspects such as age, pregnancy, lactation, and dietetic inhibitors or enhancers of absorption. Phytates and oxalates are the most prominent inhibitors of calcium in plant foods. Therefore, plant-based consumers must consider these factors in order to choose foods that are low in inhibitors and high in calcium like soy derived products fortified with calcium. Furthermore, serum vitamin D levels must be within optimum range for the body to absorb calcium. Calcium excretion may also be increased by excessive intake of sodium, animal protein, caffeine, and phosphorus (e.g., phosphoric acid in carbonated beverages), thus decreasing calcium body levels [80].

An emerging fermentation technique that consists of mixed cultures can be of particular interest. A mixed culture of *Lactobacillus acidophilus* and *Lactobacillus plantarum* resulted more effective than fermentation of the individual strains in eliminating phytic acid and trypsin inhibitors in cowpea. Similarly, a mixed culture of *Streptococcus thermophilus* and *Bifidobacterium infantis* dramatically decreased phytic acid and saponin levels in soy. It was further found that a mixed *Saccharomyces boulardii* and *Lactobacillus plantarum* fermentation increased calcium bioavailability approximately six-fold compared to the mono-culture fermentation [47]. On another note, Spirulina (*Arthrospira platensis*) is reported to contain calcium as high as 180% compared to milk [57], which can also be potentially used to increase calcium intake (Table 2).

As for the food industry, it is important to take into account calcium inhibitors and enhancers when developing plant-based products and fortified alternatives as well as other nutrients, such as vitamin D that also need to be present in order to absorb calcium. Moreover, optimization of fermentation techniques could increase calcium bioavailability.

## 5. Labeling Regulations

The disruption of the market by vegetarian and vegan products has brought attention to the need for labeling regulations for these products. In 2018, the European Commission approved the “Mandatory food labelling Non-Vegetarian/Vegetarian/Vegan” initiative. This initiative proposed laws mandating the use of pictorial labels on all food products to help vegetarians and vegans identify suitable food products and reduce ambiguity [93]. Currently, there is no legal definition to label a product as vegetarian or vegan food in the EU, and the terms are being defined at the EU level. The use of meat terms when labeling plant-based meat alternatives is unclear because there are few legal names for meat products, with a few exceptions. Meat products regulation contains only general sales descriptions, but currently no different language versions of terms for meat products like sausage, prosciutto or steak. Therefore, establishing a list of protected terms for meat products (where plant-based meat alternatives would not be included) in the EU is challenging [27].

There have been proposals to better regulate labels on meat-free plant-based alternatives to avoid confusion among consumers. A recent example is the proposal to the European Parliament to reserve the use of terms like “burger”, “sausage”, “scallop” or “steak” exclusively for products containing meat [94]. In opposition to these proposals, it has been argued that current labeling is not misleading and the use of meat terms are useful to set up expectations. Certain labels like “sausage” or “nuggets” inform the consumer the type of meat the product is trying to mimic and the kind of sensory properties that should be expected from that product.

Dairy alternatives, especially milk, have harsher legislation barriers than meat alternative products, concerning the use of dairy terms. To use such names, like “milk”, a product must comply with the legal description of what “milk” is. Because milk is described as “mammary secretion obtained from one or more milking”, plant-based milk alternatives cannot be described (labeled) as “milk”. Under these laws, plant-based products cannot use protected dairy terms laid down in Annex VII of Regulation (EU) 1308/2013 [46]. Contrary to meat products, dairy contains a long list of reserved names in different languages, exclusively for milk products, including whey, cream, butter, buttermilk, butter oil, caseins, anhydrous milk fat (AMF), cheese, yogurt, kefir, koumiss, viili/fil, smetana, fil, rjaženka and rūgušpiens. To complicate things further, EU Member states have approved exemptions in their national language, allowing certain terms that are not allowed in other countries [46]. Outside the EU in Australia, for example, the term “soy milk” is allowed.

Such heterogeneity of regulations among states of the EU, as well as between countries can create a climate of uncertainty and result in a stifling of investment in innovation [95].

## 6. Product Innovations for Plant-Based Diets

### 6.1. Cultured Meat

Cultured meat has been presented earlier as one of the newest alternatives to livestock meat. This alternative has been proposed as a more sustainable source of meat than livestock, although the environmental impact of large-scale cultured meat production is still unclear. Cultured meat has been estimated to reduce land use by 99% for its production compared to livestock meat production. Nevertheless, other energy needs and a strict hygiene installation required for the production of cultured meat makes it inefficient in terms of energy, water and feedstock expenditure [34]. Energy cost and CO₂ derived from cultured meat production has been estimated to be worse than cattle in the very long term [35], as long as we continue to use carbon-based energy sources.

An important innovation in this alternative is the capacity to alter the nutritional profile of the meat by altering the medium. This can be done by adding nutrients, for example vitamins, or modifying the fatty acid profile of the meat. Co-culturing the cells with adipocytes could increase tissue fat and therefore help modify the flavor of the final product [36].

Therefore, cultured meat is a promising alternative for its nutritional and sensory properties, further investigation is warranted to reduce the energy costs and environmental impact cultured meat currently requires for its production.

### 6.2. Milk and Dairy Alternatives

The main challenges of milk alternatives are to provide a desirable and acceptable sensory experience for consumers and to match the nutritional value of milk. To compensate for possible nutritional deficiencies, fortification with vitamins and amino acids is applied to these products. Furthermore, fermentation of milk alternatives improves sensory perception because it decreases the beany flavor of plant materials and provides desirable volatile flavors [47]. The addition of a starter culture to plant-based beverages is also used in the production of vegan or plant-based yogurts. Probiotic strains can be added to soy-based yogurts, and these strains can compete better with starter cultures in a soy beverage than in cows’ milk [96].

Fermentation with two or more microorganisms can improve plant protein solubility and amino acid composition and availability. For example, *Bifidobacterium* significantly increased the protein content of soy-based drinks. Moreover, fermentation of soybean with *Lactobacillus plantarum* resulted in an increase of essential amino acids such as lysine. Furthermore, co-fermentation of peanut using *Lactobacillus acidophilus* and *Lactobacillus plantarum* significantly increased the total protein and lysine, methionine and tryptophan contents compared to those of the corresponding mono-culture fermentations. Spontaneous co-fermentation of strains originating from cowpea and chickpea improved methionine levels. However, in other cases, mixed-culture fermentation appeared inferior to mono-culture processes [47].

In Asia, cheese analogs have been around since the 1500s. Fermented tofu has been used as a substitute because its strong aroma reminds of mold-ripened cheeses like Roquefort or Camembert. Although, it does not melt, it can be easily spread. To produce this alternative cheese, tofu cubes are inoculated with a special mold and left to ripe in a warm environment to let the mycelium grow [97].

In Western countries, the production of cheese analogs involves the use of fat and/or protein sources other than those from cow’s milk, together with flavors that resemble as closely as possible those of the original product. It is important to note that cheese analogs can completely exclude milk and milk products (vegan) or partially contain milk or milk components (e.g., casein, butter oil, etc.) together with coconut or soybean extract [48]. In cheese analogs, the milk protein and milk fat are partly or totally replaced by vegetable proteins (i.e., peanut protein, soybean protein) and vegetable fats and oils (i.e., partly hydrogenated vegetable fat like soybean, palm, etc.). Cheese analogs have been consumed for decades, mainly used in pizza as cheaper alternatives to cut production costs. Still, the difficulties of reproducing the unique flavors of different types of cheese is what has prevented the use of cheese analogs as “cheese board” products for many years [48].

In recent years, innovations in vegan cheese recipes and preparation methods have greatly improved the sensory properties of these products. Soy, nuts, coconuts, tapioca, and even potatoes are used to prepare them. Vegan Parmesan cheese can be prepared grinding nuts and nutritional yeast together. Cashews can be soaked in rejuvelac (fermented grain beverage) to obtain vegan Mozzarella [98]. Arrowroot and cassava have also been used to make commercial vegan cheese because they provide melting and stretching properties, which are hard to replicate without casein [99].

### 6.3. Egg Alternatives

Egg alternatives are used instead of eggs in recipes by consumers following a vegan diet or individuals with egg allergy. While other products such as meat alternatives aim to replicate the sensory qualities and experience of consuming the original product, the main objective of the egg alternative is to replace the functional properties of egg protein (solubility, emulsification, foaming and gelling) for backing and cooking. “Vegan mayonnaise” is the most tested product in studies on the effectiveness of vegetable ingredients to substitute the egg’s properties. Preparation of cholesterol-free and modified lipid versions of such products is also of interest.

Söderberg [51] investigated the use of soy and pea protein to substitute egg protein and found that both soy and pea protein have properties that are similar to that of egg, although pea protein showed poor gelling properties. Furthermore, according to the protein digestibility-corrected amino acid score (PDCAAS), soy was found to have similar protein quality to egg, but pea protein was found to be incomplete. In order for pea protein to become complete, the complementarity concept in terms of amino acid composition between different plant protein sources may be used (see Section 4.3.1.). Another aspect that should be taken into account is that soy and pea protein have distinct flavors due to their content in saponins, ketones and aldehyde compounds. These flavors are often described as “beany” or “green” and can be off-putting to the consumer.

Garcia et al. [52] formulated a mayonnaise containing rice bran oil and soy protein concentrate which received an overall low acceptance rating among consumers. Only after further flavors were added acceptance increased considerably. In addition, the intent to purchase significantly increased when consumers were informed of the potential benefits of the new formulation.

Vegan mayonnaise has also been prepared using soy milk, xanthan gum and guar gum as stabilizers [53] and using arabic gum alone [54]. Other vegetable proteins from soy, sunflower, pea, tomato seed, wheat, white lupin and faba bean have been successfully tested to stabilize oil-in-water emulsions [53]. To substitute eggs in baking and cooking, ingredients as varied as apple sauce, aquafaba, flax seeds, tofu, ripe bananas and tapioca starch can be used [100]. Although there is an ample list of ingredients that can emulate eggs when cooking, the nutritional value of these ingredients must be taken into account. Protein rich foods like legumes can offer similar protein content, while other ingredients contain only marginal protein. If protein content differs notably, consumers must be mindful to avoid nutritional deficiencies from regular consumption of egg alternatives with insufficient protein content.

### 6.4. Microalgae

Microalgal biomass has been consumed by the indigenous populations to survive during extreme food shortages as, depending on the species, can contain up to 70% protein or 40% of marine oils [101]. Protein of algae contains all essential amino acids, with some species showing comparable amino acid profiles to soybean and eggs. However, digestibility and bioavailability are also major factors as the cell wall interferes in the utilization of nutrients. To increase bioavailability of microalgae proteins a pretreatment can help disrupt the cell wall [56]. *Schizochytrium sp* is a good source of the omega-3 fatty acid DHA and its oil mechanically extracted has been accepted as a new food by the EFSA [102]. This can be included in infant formula, follow-on formula and other products.

### 6.5. Fish Alternatives

Common fish alternatives are made with tofu and seitan (wheat gluten) to which soy sauce, miso paste or algae are added to provide the sea-like taste. Furthermore, some microalgae are sustainable sources of protein while others are sources of omega-3 and could contribute to the dietary intake of EPA and DHA [56].

The issue with some current fish alternatives is that they do not provide any of the nutritional benefits of fish and seafood consumption. In this regard, neither tofu nor seitan are sources of EPA and DHA, and other fish alternatives made from vegetables do not provide protein or omega-3 FAs. In vegan and vegetarian recipes, mushrooms are sometimes used as substitutes for seafood, peeled and marinated tomatoes and carrots have been proposed as marinated tuna, and salmon, respectively. Although these are clever imitations that emulate sensory properties, they should be improved to achieve an adequate nutritional profile.

## 7. Health Opportunities of Plant-Based Diets

Plant-based diets have been shown to be cardioprotective and beneficial for certain diseases such as type 2 diabetes, obesity and hypertension [69,103,104,105,106,107]. These diets might be useful to prevent and treat certain diseases as they involve mechanisms that reduce cardiovascular risk factors like abdominal obesity, blood pressure, lipid profile and blood glucose [69,104,105,106]. However, conflicting results have been found regarding the impact of a vegetarian diet on high density lipoprotein-cholesterol (HDL-C) and low density lipoprotein-cholesterol (LDL-C). For example, some studies have found no difference in plasma HDL-C levels between vegetarians and omnivorous diets. Other studies of vegetarian populations found lower total cholesterol and LDL-C in a study population, which is associated to reduction of cardiovascular risk, but also lower HDL-C [106].

Plant-based eaters regularly consume a variety of fruits, vegetables, whole grains, legumes, and nuts. Additionally, they consume less saturated fat while substituting it for polyunsaturated fat [104,105]. In particular, vegan diets have been shown to be the most beneficial according to some authors [105,106], the EPIC-Oxford study, and Adventists Health Study-2 (AHS-2) [108], which revealed that vegans ate the most fiber, the least total fat and saturated fat, and had the healthiest body weight and cholesterol levels compared to omnivores and vegetarians [69,108].

In relation to diabetes and obesity, plant-based diets have shown beneficial effects as they include foods that contain high amounts of fiber, antioxidants, magnesium and phytochemicals, all of which have shown to increase insulin sensitivity and glycemic control [104,105,109]. Additionally, obesity is one of the main causes of type 2 diabetes. One of the reasons plant-based diets might be effective at preventing and treating this condition is because these foods promote weight loss, reduce the energy density of foods and promote satiety, thus reducing insulin resistance [104]. In this regard, plant-based dietary patterns are associated with lower body mass index (BMI; calculated as kg/m^2^) [69,110], as well as reduced inflammation [106], compared with patterns where animal products are abundant. Moreover, studies have also shown that saturated fat, which is mainly found in animal-derived foods, contributes to the increase of insulin resistance by accumulation of toxic fat metabolites in hepatic and skeletal muscle cells, impairing insulin signaling and thus decreasing glucose uptake [104].

However, with respect to diabetes and obesity, in order to benefit from the therapeutic effects of a plant-based diet, a healthy dietary pattern must be followed [104,105]. This statement is evidenced by the “Seguimiento Universidad de Navarra” (SUN) cohort, which is a large prospective cohort study of relatively young adults, better conformity with a healthy vegetarian diet was associated with a reduced long-term risk of overweight/obesity, whereas no consistent trend was found for a food pattern that emphasized less-healthy plant foods [110,111].

On another note, food microbiome has been a topic of interest regarding diabetes and obesity as it has been shown that food microbiome interactions can improve insulin sensitivity and inflammation response [104,105]. For example, dietary fiber, which is found only in plant foods, may play a key role in this process as it modulates postprandial glucose response and is fermented by intestinal bacteria to produce short-chain fatty acids, which also improve insulin signaling and glucose response while modulating host’s inflammatory response [104,106]. Accordingly, vegetarian diets influence food microbiome interactions providing further benefits. A study of 144 vegetarians, 105 vegans, and an equal number of matched omnivores found that vegan and vegetarian diets produced a significant shift in the gut microbiota, with a significant reduction in the vegan subjects of *Enterobacteriaceae*, which is a family of bacteria implicated in triggering low-grade inflammation. Another study of six obese subjects with diabetes and/or hypertension who followed a vegan diet for one month found improved blood glucose levels and reduced body weight, a decrease in *Enterobacteriaceae* and *Firmicutes* (associated with Western diets and low-grade inflammation) with a significant increase in *Bacteroidetes* (associated with low calorie and vegetarian diets). Therefore, high levels of fiber in vegan and vegetarian diets may contribute to reduce levels of inflammation and decrease risk for metabolic disease and obesity [106].

In regard to hypertension, clinical trials generally support the finding that a vegetarian diet may reduce it. According to the AHS-2 study [108], vegetarian diets may be associated with a reduction in both systolic and diastolic blood pressure when compared to omnivorous diets. In this study, it was found that vegetarians, and especially vegans, had lower systolic and diastolic blood pressure and lower hypertension prevalence than the omnivores in the cohort. In addition, lower LDL-C, lower total cholesterol, and reduced risk for obesity and diabetes was found among the participants [106,108].

More investigations are needed about plant-based diets regarding chronic diseases and the different benefits presented by gender and population groups [106], and further investigations need to be done in order to confirm therapeutic effects in other diseases such as colon cancer and ischemic heart disease.

## 8. Conclusions

The number of consumers who are reducing their intake of food from animal origin is increasing globally due to many reasons and this involves a growing market of plant-based products. Consumers demand products that are sustainable, palatable, safe, nutritious, available, and affordable. There are many issues that should be considered when planning new sources and ingredients. In this regard, the production of alternatives to meat, such as cultured meat, has a great potential but still needs optimization. Other biotechnology processes including microalgae culture, fermentation or addition of microorganisms, such as those that are producers of vitamin B_12_, have also a great potential. It should be emphasized that these plant-based products may be applicable to subjects with special needs, e.g., due to allergy to compounds present in food from animal origin or chronic diseases that are known to revert by reducing animal food and increasing vegetables intake. Finally, being aware of protecting the planet means that plants, animals and humans are part of it and a healthy planet should be compatible with a healthier human being.

## Figures and Tables

**Table 1:** Food products for vegetarian and vegan consumers.

Products	Definition and Sources	Advantages	Disadvantages	References
Plant-Based Protein Products	Protein rich products from plant foods: soy (tofu, tempeh, texturized soy protein); wheat gluten (seitan); legumes (pea, lentil, lupine, chickpea); seeds (rapeseed, canola).	Perception of being healthier and more sustainable than meat. Higher acceptance when it resembles processed meat (burgers, sausages, nuggets). More familiar to consumers than mycoprotein or cultured meat. Lowest environmental impact of all meat alternatives. Products have been on the market for decades.	Meat consumption is highly ingrained in culture; willingness to stop or reduce meat consumption is low. Taste, texture and appearance often unappealing to meat consumers. Inconvenient to find in stores, high prices and difficulty in cooking. Push to ban meat terms for meat alternative products.	Kumar et al. [18] Malav et al. [19] Kyriakopoulou et al. [20] Aschemman-Witzel et al. [11] Pohjolanien et al. [21] Piazza et al. [22] Michel et al. [23] Corrin and Papadopoulos [24] Bryant [25] Koning et al. [26] Clune et al. [5] Soret et al. [6] Sanchez-Sabate and Sabaté [7] Carreño and Dolle [27]
Mycoprotein	Product obtained from fermentation of the fungus *Fusarium venenatum*.	Land use is substantially lower than that used for conventional meat production.	Estimated global warming impact higher than chicken, pork and soy-based alternatives.	Finnigan [28] Filho [29] Smetana [30]
Cultured Meat	Meat produced from the growth of cultured animal cells in a nutrient rich medium. Livestock cells	Highest resemblance to original livestock meat. Land use estimated 99% lower than livestock meat production. Minimal use of animals for meat production.	Perception of being unnatural worries about safety. Higher CO_2_ emissions than meat, inefficient water and feedstock expenditure. Requirements according to novel food regulation.	Chriki and Hocquette [31] Bryant and Barnett [32] Siegrist et al. [33] Alexander et al. [34] Lynch [35] Bhat and Fayaz [36]
Plant-Based Milk Alternatives	Water-soluble extracts from plant material broken down and extracted in water for further homogenization: legumes (chickpeas, soybeans); cereals (oats, rice); pseudo-cereals (quinoa, teff, amaranth); nuts (almonds, cashew nuts, hazelnuts, walnuts, coconut); seeds (sesame, sunflower).	Perception of being more sustainable. Positive perception of taste when flavored. Fermentation can improve nutritional bioavailability and sensory properties. More sustainable than cow’s milk (except almond milk).	Bland taste when not flavored. Concerns of added sugars and artificial sweeteners. Regulatory barriers to use protected dairy terms. Almond milk has higher environmental impact due to irrigation.	Sethi [37] Silva et al. [15] Palacios et al. [38] Palacios et al. [39] Villegas et al. [40] Schyver and Smith [41] Schiano et al. [42] McCarthy et al. [43] Ritchie et al. [44] Grant and Hicks [45] Leialohilani and de Boer [46] Tangyu et al. [47]
Cheese Alternatives	Products from milk protein and milk fat that are partially or totally replaced by vegetable proteins (i.e., peanut or soybean protein) and vegetable fats and oils (i.e., partly hydrogenated vegetable fat like soybean, palm, etc.): soy, nuts, coconut, tapioca, nutritional yeast.	High quality protein when soy is used. Possibility to alter lipid profile and reduce saturated fat content. Cost reduction for food manufacturers when substituting cheese as an ingredient for cheaper alternatives. Longer shelf life.	Some cheese alternatives are not nutritionally equivalent and can lack relevant nutrients if not added. Palm oil used for cheese alternatives can come from non-sustainable sources. Some products have high saturated fat content from coconut and palm oil.	Bachmann [48] Gesteiro et al. [49] Saswattecha et al. [50]
Egg Alternatives	Products, ingredients or mix of ingredients used to substitute egg: xanthan, guar, arabic gums; proteins from soy, sunflower, pea, tomato seed, wheat, white lupin and faba bean; applesauce, aquafaba, flax seeds, tofu, ripe bananas and tapioca starch.	Able to imitate the functional properties of egg protein (solubility, emulsification, foaming and gelling) for backing and cooking. Allows for preparation of cholesterol-free products (e.g., mayonnaise).	Soy and pea used as egg substitute can give an unpleasant flavor to the final product.	Söderberg [51] Garcia et al. [52] Nikzade et al. [53] Ali and EL Said [54]
Fish Alternatives	Products, ingredients or mix of ingredients used to substitute fish and seafood: soy and wheat, gluten, algae, mushrooms, vegetables.	Does not contribute to overfishing.	Most alternatives are nutritionally deficient in protein and essential fatty acids EPA and DHA.	Caporgno and Mathys [55] Malchira et al. [56]
Microalgae	Microscopic algae, ingredients or products, rich in protein, carbohydrates, lipids and other bioactive compounds: *Chlorella sp, Arthrospira sp. Schizochytrium sp.*	Requires less land use than livestock. Does not compete for agricultural land. Helps fix CO₂. Source of EPA and DHA.	Regulatory issues if GMO microalgae are used to improve composition. Ecological and environmental risks of GMO microalgae must be properly assessed. Acceptance might be low due to marine taste.	Koyande et al. [57] Charles et al. [58] Caporgno and Mathys [55]

EPA, Eicosapentaenoic acid; DHA, docosahexanoid acid; GMO, genetically modified organism.

**Table 2:** Current vs. new nutrient fortifications in plant-based foods.

Nutrient	Current Sources and Processes	New Sources and Fortified Products	References
Protein	Plant-based products with plant protein: soy, chickpea, other legumes.	Microbial fermentation (mono- and mixed-cultured bacteria) to increase protein. Microalgae as a source of protein. Development of products with all essential amino acids and decreased inhibitors to improve bioavailability.	Tangyu et al. [47] Malchira et al. [56]
Vitamin B_12_	Food fortification: breakfast cereals, non-dairy milk and yogurt alternatives among others.	Fortification by natural vitamin B12-producing microorganisms through lactic fermentation. Lupin fermentation to make “lupin tempeh”. Hydroponic cultivation technique, which allows crops to grow directly in water enriched with B12.	Gallego-Narbón et al. [75,76] Tangyu et al. [47] Watanabe et al. [77] Wolkers-Rooijackers et al. [78]
Vitamin D	Food fortification: milk, eggs, plant-based drinks, breakfast cereals, mushrooms. Lichens D_3_ as supplements.	Vitamin D-biofortified eggs. Ultraviolet (UV) irradiation of mushrooms and baker’s yeast. Lichens: the use in foods needs to be further investigated and evaluated.	García-Maldonado et al. [74] Vessanto et al. [69] Cardwell et al. [79] Hever [80]
Iron	Food fortification: salt, sugar, cereal-based products, milk, and other dairy products.	Biofortification. Ferritin content enrichment. Phytic acid reduction (e-g. adding phytases during baking). Microencapsulation of the iron fortificant before adding it to the food vehicle. Ascorbic acid addition.	Blanco-Rojo and Vaquero [81] Gallego-Narbon et al. [82] Shubham et al. [83]
Omega-3	ALA sources: flaxseeds, hempseeds, chia seeds, leafy green vegetables, walnuts, wheat germ, and their derived oils.	Cultured microalgae. Biofortification of foods like dairy and eggs by incorporating fish oil or algal to cows’ and hens’ feed.	Salvador et al. [84] Hever [80] García Maldonado et al. [74] Charles et al. [58] Stamey et al. [85]
Calcium	Food fortification: plant-based drinks and breakfast cereals.	Fermentation technique: mixed culture of bacteria. Consumption of Spirulina (Arthrospira sp)	Tangyu et al. [47] Koyande et al. [57]

ALA, alpha-linolenic acid.
